# A simulated ‘sandbox’ for exploring the modifiable areal unit problem in aggregation and disaggregation

**DOI:** 10.1038/s41597-024-03061-1

**Published:** 2024-02-24

**Authors:** Jeremiah J. Nieves, Andrea E. Gaughan, Forrest R. Stevens, Greg Yetman, Andreas Gros

**Affiliations:** 1 University of Glasgow, School of Geographical & Earth Sciences, Glasgow, UK; 2 University of Louisville, Dept. of Geographic and Environmental Sciences, Louisville, USA; 3 Center for International Earth Science Information Network (CIESIN), University of Columbia, Columbia, USA; 4 Vibrant Planet PBC, Nevada, USA

**Keywords:** Geography, Research data, Interdisciplinary studies

## Abstract

We present a spatial testbed of simulated boundary data based on a set of very high-resolution census-based areal units surrounding Guadalajara, Mexico. From these input areal units, we simulated 10 levels of spatial resolutions, ranging from levels with 5,515–52,388 units and 100 simulated zonal configurations for each level – totalling 1,000 simulated sets of areal units. These data facilitate interrogating various realizations of the data and the effects of the spatial coarseness and zonal configurations, the Modifiable Areal Unit Problem (MAUP), on applications such as model training, model prediction, disaggregation, and aggregation processes. Further, these data can facilitate the production of spatially explicit, non-parametric estimates of confidence intervals via bootstrapping. We provide a pre-processed version of these 1,000 simulated sets of areal units, meta- and summary data to assist in their use, and a code notebook with the means to alter and/or reproduce these data.

## Background & Summary

Decision-making criteria regarding the spatial scale and zonation of areal units has a fundamental impact on the nature of geographic spatial analysis^1–7^. While this phenomenon has been acknowledged in the geographic literature for decades^1–6^, being cognizant of the implications in how underlying data is constructed matters for any field, with particularly useful examples noted for demographic^8^, health^9,10^, urban^11^, and ecological^12^ applications. Considering that no rule set or agreed upon standards currently exist for areal aggregation in spatial analysis^1,2,4,5^, it is critical to determine the underlying rationale for a given spatial resolution in geographical analysis as the sensitivity of associated outcomes is tied directly to the decision-making criteria of model development and the underlying characteristics of the data^1,2,5^.

Notably, the modifiable areal unit problem (MAUP) is a well-known issue in the geographical literature and describes how sensitive analytical results are to the size and configuration of the areal units informing the analysis^1–7^. Different spatial scales chosen for the aggregation of the data can result in different outputs. Similarly, *how* the data is aggregated can also impact the spatial analysis and modelling outputs^1–3,13,14^. Recent work highlights considerations of spatial properties associated with the MAUP effect on both the underlying data and the underlying processes, also drawing attention to differences of fitting a model locally or globally^15^. The influence of MAUP is a result of spatially varying processes and distributions of data that can, at least partially, be assessed through model estimates or statistical properties associated with a given decision criteria and should be considered an inherent aspect of geographical analysis^1–5,15^. Geographical analyses that depend on spatial units for analysis require clear articulation regarding decision criteria for data choice, manipulation, and aggregation processes. Even with specific spatial scales rationalized, reproducibility and replicability can be challenging due to MAUP properties^15^.

Previous works have used nested hierarchical sets of areal units, e.g., census-based subdivisions, to provide calculations tied to the various units. However, those units remain only one potential zonal configuration at the given spatial scale, or resolution, used in analysis^1,16–18^. Relative to the number of works utilising spatial analysis in some form, very few works have examined simulated aggregations, which may be better able to capture the range of potential scales and zonal arrangements that a fixed area could conceivably be partitioned into. Those that did were limited in their extent and complexity due to: i) computational constraints of the time, ii) scope of the research question, and or iii) their comparability was limited due to different areal units and study areas used or method specific conclusions^2,4,13,19–21^.

We explore such challenges with gridded population data, a product derived from a modelling process that has become more prevalent in applied contexts since the 1990s^22^. The use of gridded population data continues to provide important and timely information on the spatial and temporal distributions of population count and density, and these data products are widely used by international agencies, governments, and academic institutions world-wide^23^. With a world population over 8 billion, and continued rapid growth, demographic changes will have significant socioeconomic, development and health impacts, radically alter land use and affect the climate change risk landscape^23–27^. Effective planning and resource allocation strategies require a strong evidence base that takes these changes, their spatial distribution and scale into account, necessitating timely measuring and mapping of population^23,26,27^. However, the demand for gridded population data products is tempered by an awareness that not all gridded products are created equal, driven by differences in the underlying model structure, assumptions, inputs, data uncertainty, and, particularly, the spatial scale and configuration of the input areal population data^22^.

Census-based disaggregative models are a modelling approach where population counts are redistributed from coarser irregular spatial resolution units to a smaller scale of standardised grid squares^28–32^. This “top-down” method of generating continuous raster surfaces of population counts and/or densities gained traction in the 1990s with the Gridded Population of the World project and dataset^28,33^. Continual advancement in method development informed by data extraction techniques (e.g. land cover, urban designations, settlement mapping) and different statistical tools (e.g. machine learning, probability estimation) has resulted in multiple, open-access global and regional data products (https://www.popgrid.org/). A good review of these different data products and an in-depth summary of their fitness for use is found in^22^. The gridded population modelling field continues to advance methods to include hybrid census techniques^34,35^, other demographic characteristics^36^, and dynamics and mobility characteristics^37^, but a base population denominator remains a vital population attribute underlying most human related data.

Recognizing there are multiple ways to spatially model population^22,30–32,35,38–42^, a widely used and contemporary method leverages the random forest (RF) algorithm^38^. RFs are a machine learning approach first described in^43^, increasing the robustness of single classification and regression tree (CARTs) predictions through an ensemble approach that combines multiple CARTs with random bagging sampling^44^. In a dasymetric population disaggregation context, countries have different numbers of available units for training and prediction along with the underlying populations having complex, non-linear, and varying relationships to the predictive covariates^45^. As such, RFs are useful given their robustness to large and small sample sizes and noise, ability to capture non-linear relationships, and minimal manual parameter adjustment.

However, in using a top-down dasymetric disaggregation approach, the gridded population outputs are trained at a coarser “source” level than the finer “target” level^31^, which creates differences in the range of population densities from source to target level and introduces potential underestimation in the dispersion of the data as well as extremes in the distribution^46^ Also noted in the literature is the tendency to overestimate population densities in urban areas while underestimate in more rural areas, a direct reflection on the unit sizes and aggregation levels that represent more highly populated areas versus not^37^. Little rigorous examination exists on how any spatial model, or aggregation/disaggregation procedure, is affected by choice of spatial resolution and zonal configuration of the areal units^1–5,13,20,21^.

Challenges persist on fine scale validation of modelled population data, the quantification of uncertainty, and any potential systemic biases that result from the combination of the input data, spatial scale and zonal configuration of such data, and the disaggregative model process. More specifically, how well do the modelled populations perform across the spatially varying characteristics of the true underlying population? Part of why these questions have not been answered is the expense, e.g., time, computation, and code, to produce multiple realisations of areal units and the lack of a standard benchmark dataset from which different approaches could be tested and compared against.

To further research production, knowledge-sharing, and engagement for modelling gridded population, we present a set of data^47^ and corresponding code for exploring relationships of scale, bias, and accuracy with census-based disaggregative population modelling. We utilise a building- to block-level population dataset in Guadalajara, Mexico to simulate 10 levels of spatial resolutions, ranging from levels with 5,515 - 52,388 units and 100 simulated zonal configurations for each level – totalling 1,000 simulated sets of areal units. These data^47^ can facilitate interrogating various realizations of the data and the effects of the spatial coarseness and zonal configurations, the MAUP, on applications such as model training, model prediction, disaggregation, and aggregation processes.

We briefly exemplify this by utilising a RF-informed dasymetric disaggregation of population counts to 100 m pixel level from various spatial resolutions and simulated zonal configurations. More broadly, these types of data (hierarchical, simulated aggregations of areal units) might be useful for testing and development in a variety of spatial statistical contexts, including those of small area estimation (SAE)^48,49^ and other spatial disaggregation approaches (e.g. post-stratification of survey^50^, or aggregation processes). Though the data^47^ we provide do not attempt to aggregate attributes other than population counts, the underlying census data could be linked with various demographic or socioeconomic attributes.

## Methods

### Study Area

The data presented here comprise the urban region of Guadalajara, Mexico and its rural surroundings. It is bounded roughly by the rectangle with corners at 19.92° N, 104.09° W, and 21.08° N, 102.95° W. This region around Guadalajara, Mexico, covers parts of the states of Jalisco and Aguascalientes and is characterised by a diverse landscape of urban areas, rural farmland, mountains, valleys, and arid plains. The city of Guadalajara, the capital of Jalisco, is centrally located within this region and is surrounded by the Sierra Madre Occidental mountain range and the Lerma River basin. To the south, the area is dominated by primarily agricultural land use, with extensive areas of farmland punctuated by small towns and village areas and cropland. Moving northward, the terrain becomes more mountainous, covered in pine and oak forests, with peaks reaching over 3,000 meters.

The source data used to produce the synthetic datasets covered by this descriptor begin with a polygonal dataset of 55,146 features covering the study area and joined to 2010 Mexico Census data counts containing a total population of 5,027,901. The spatial and 2010 census data originate from the National Institute of Statistics, Geography and Informatics (INEGI) of Mexico, and are of mixed spatial resolution resulting from a bifurcated process of census data aggregation. Areas of more dense population are covered by small polygons, hereafter simply “units,” representing blocks or even buildings and correspond to administrative unit “Level 5,” known as the “*manzana*” level. Areas of less dense population are covered by coarser, Thiessen polygons, created from INEGI microdata centroids representing administrative unit “Level 3,” or “*localidades*.” These units are areas with populations under 5,000 people total^51^.

**Fig. 1:**
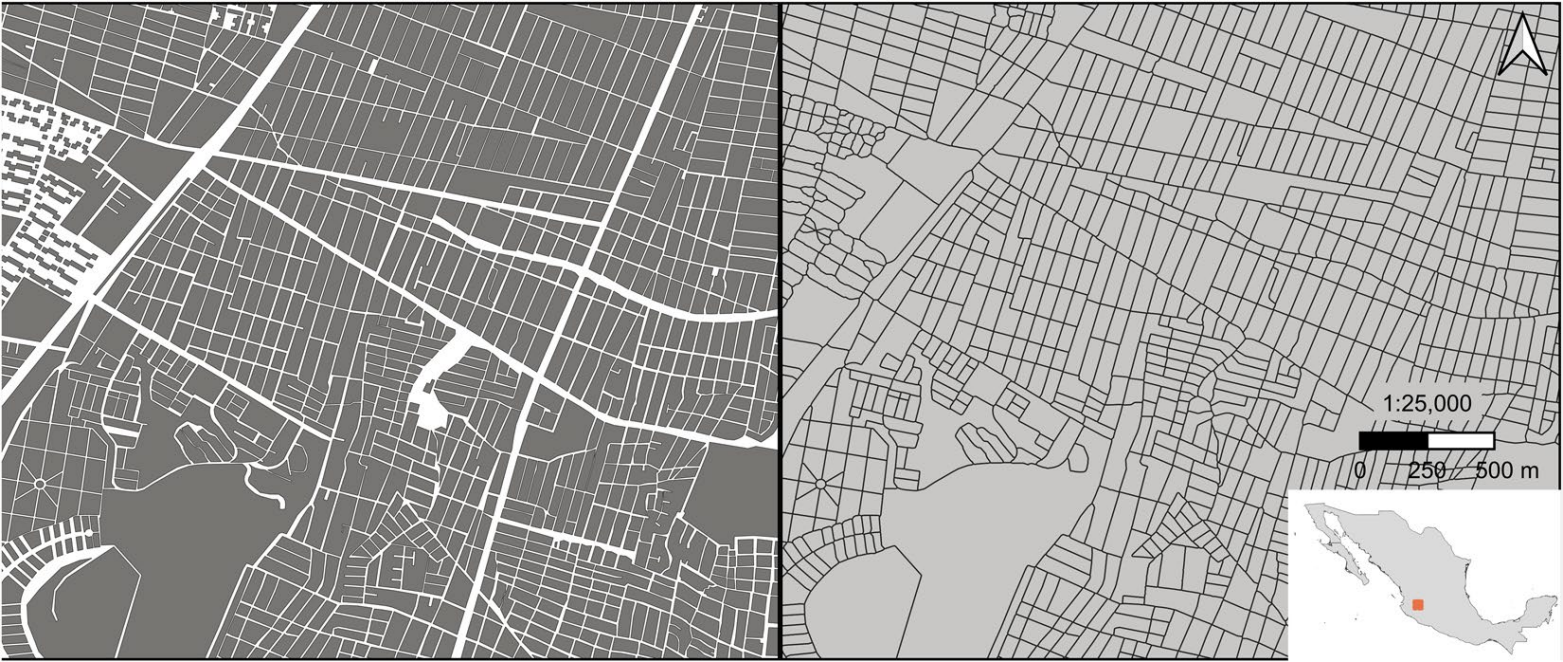
Example of the original high-resolution census-based, polygonal data with streets, water bodies, and other open spaces left as “no data” (left, shown as white space) and the same polygons after morphological tessellation (right) with the location of the study area in Mexico given in the inset map in the lower right. Each aggregated unit was then joined with its total population count corresponding to the 2010 census. These joined *localidades* and *manzana* data for the study region represent the base data product from which all previous syntheses were produced.

The very high-resolution data contained within higher population density regions contain street gaps or boundaries between units, which for the sake of uniformity with typical contiguous census data representations used in common applications, we removed prior to any further processing. The goal was to create shared borders by removing the imposed street network and open data within settlement agglomerations and exclude areas of no data. To rectify this, the polygons, representing the units, were tessellated using a morphological Voronoi tessellation executed with the package momepy^52^ in Python^53^. This expanded the polygons beyond the road gaps to where they now bordered all their nearest polygons, following the Voronoi tessellation logic (Fig. 1). These were the data that were then aggregated in the simulations and subsequently used in the population modelling.

**Fig. 2:**
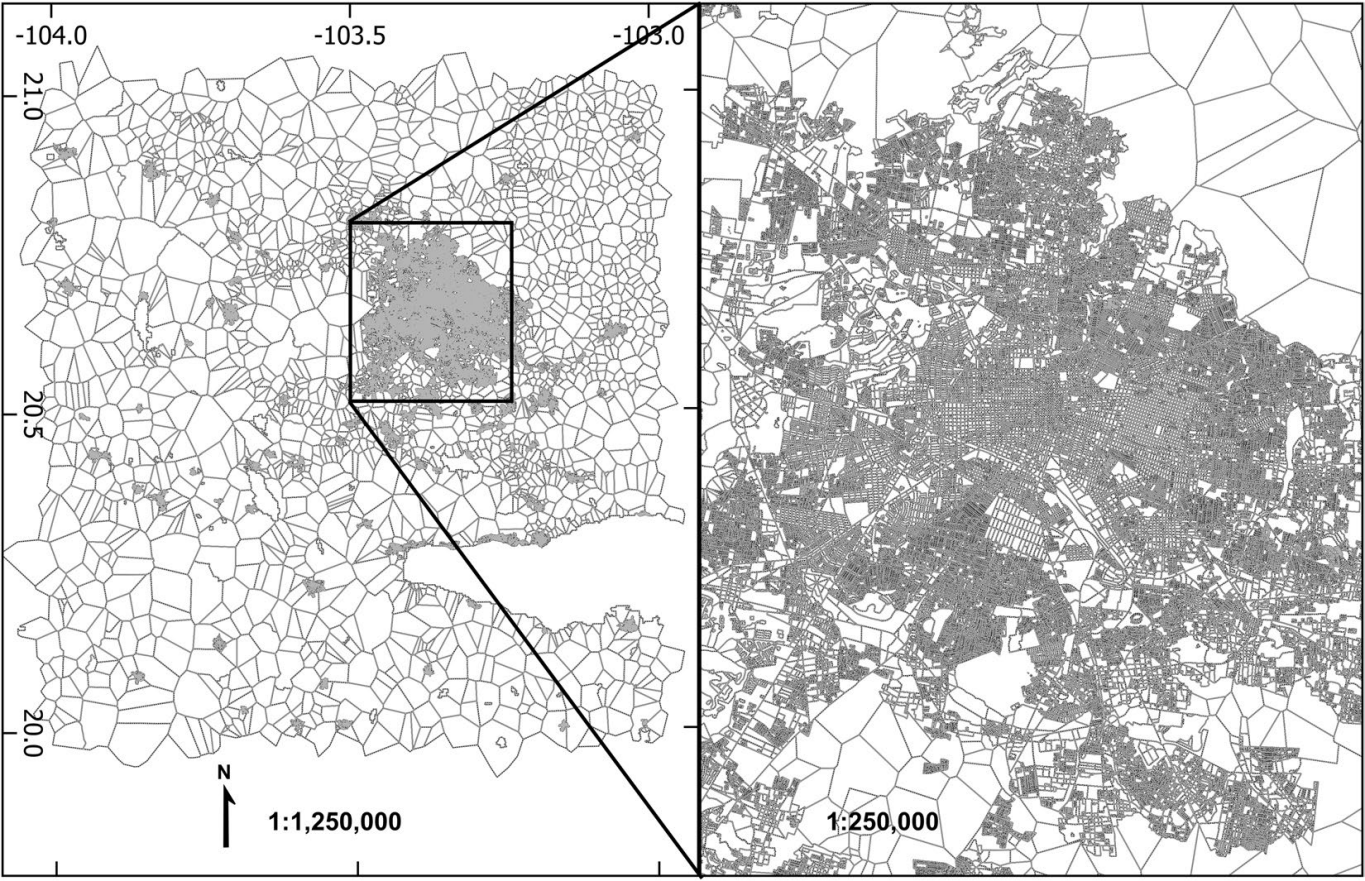
General overview of the entire polygonal dataset in the Guadalajara, Mexico study area.

As produced, the final combined population census data consisted of 55,146 polygons with an average spatial resolution (ASR) of 0.461 km. The range of spatial areas for the produced units was 29.52 m^2^ at minimum to a maximum of 6.88 × 107 m^2^ (Q1: 2919.95, Median: 5213.63, Q3: 9526.25). An overview of the dataset and the study area is shown in Fig. 2.

### Simulation Methods

#### Simulated Areal Population Data for Disaggregation

**Fig. 3:**
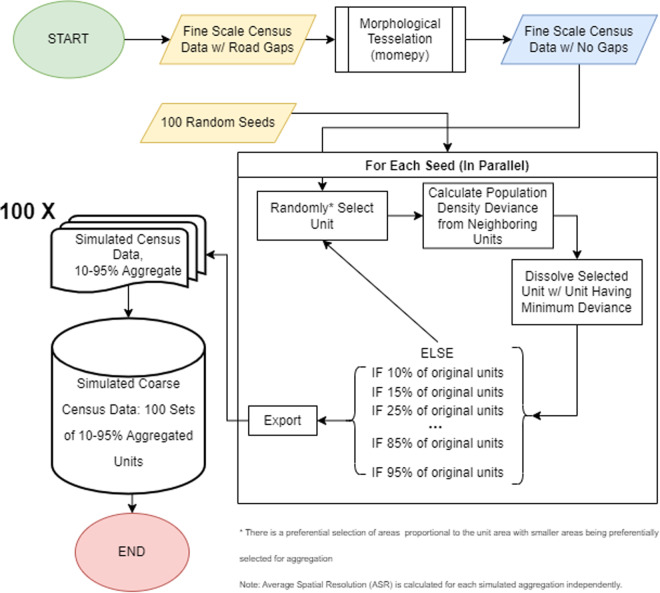
Procedure diagram of the aggregation procedure to create simulated sets of areal population data.

Since we wished to withhold the original fine scale areal population data for validation and calculation of error metrics, we needed to aggregate the areal data into datasets having a coarser spatial resolution. That is, we needed to create simulated coarsened, hereafter simply “coarsened”, areal population data sets. We created the coarsened data sets through a simulated aggregation procedure (Fig. 3) that selected a spatial unit quasi-randomly, i.e., with preference for units with smaller area, and then dissolved it with the neighbouring unit that has the most similar, average population density. The population counts of the two dissolved units were summed before moving to the next quasi-random unit selection and dissolving iteration. This iterative procedure continued until the desired number of aggregate units was met.

We say “quasi-random” as the selection was based upon probabilities defined by an exponential curve over the distribution of polygon areas. Specifically, Eq. 1 describes the probability of selection for a given unit i.

1 $$P{(selection)}_{i}=\frac{\frac{1}{(1+(are{a}_{i}-{\min }\,(area))/{({\max }(area)-{\min }(area))}^{\rho }}}{{\sum }_{i=1}^{n}\frac{1}{(1+(are{a}_{i}-{\min }\,(area))/{({\max }(area)-{\min }(area))}^{\rho }}}$$

This resulted in a preferential sampling of smaller, i.e. more urbanised, polygons for merging. We determined this to be appropriate as the majority of population and polygons are located in urbanised areas and we did not want a scenario where the majority of less densely populated areas, typically characterised by larger polygons, were always aggregated firstly. We determined the scale factor *ρ* to use in defining the probability curve based upon trial and error. We selected *ρ* = 4 as providing a balanced mix of more densely populated and less densely populated polygons being selected for merging, but this could be modified in the provided code to produce different behaviour. The merging criteria between any two units was to minimise the loss in variability of the population density values.

We simulated the coarsened areal population datasets across 100 random seeds, i.e., numerous random starting points, used for determining sampling. We determined that using 100 seeds, i.e. producing 100 different simulation trajectories, was appropriate, based on convergence behaviour and for users to be able to carry out procedures such as estimating non-parametrically bootstrapped confidence intervals. These simulations were done in 5 percent, i.e. 2,757 areal unit, increments resulting in coarsened data with 95, 90, 85,…10 percent of the original areal units. For clarity, by iterative, we mean that, for a given seed, the 90 percent simulation derives from the 95 percent simulation, the 85 percent simulation from the 90 percent simulation, and so on. In total, this resulted in 1,800 coarsened datasets – 18 (corresponding from 95 - 10 percent) per seed or 100 per target number of coarsened units.

**Fig. 4:**
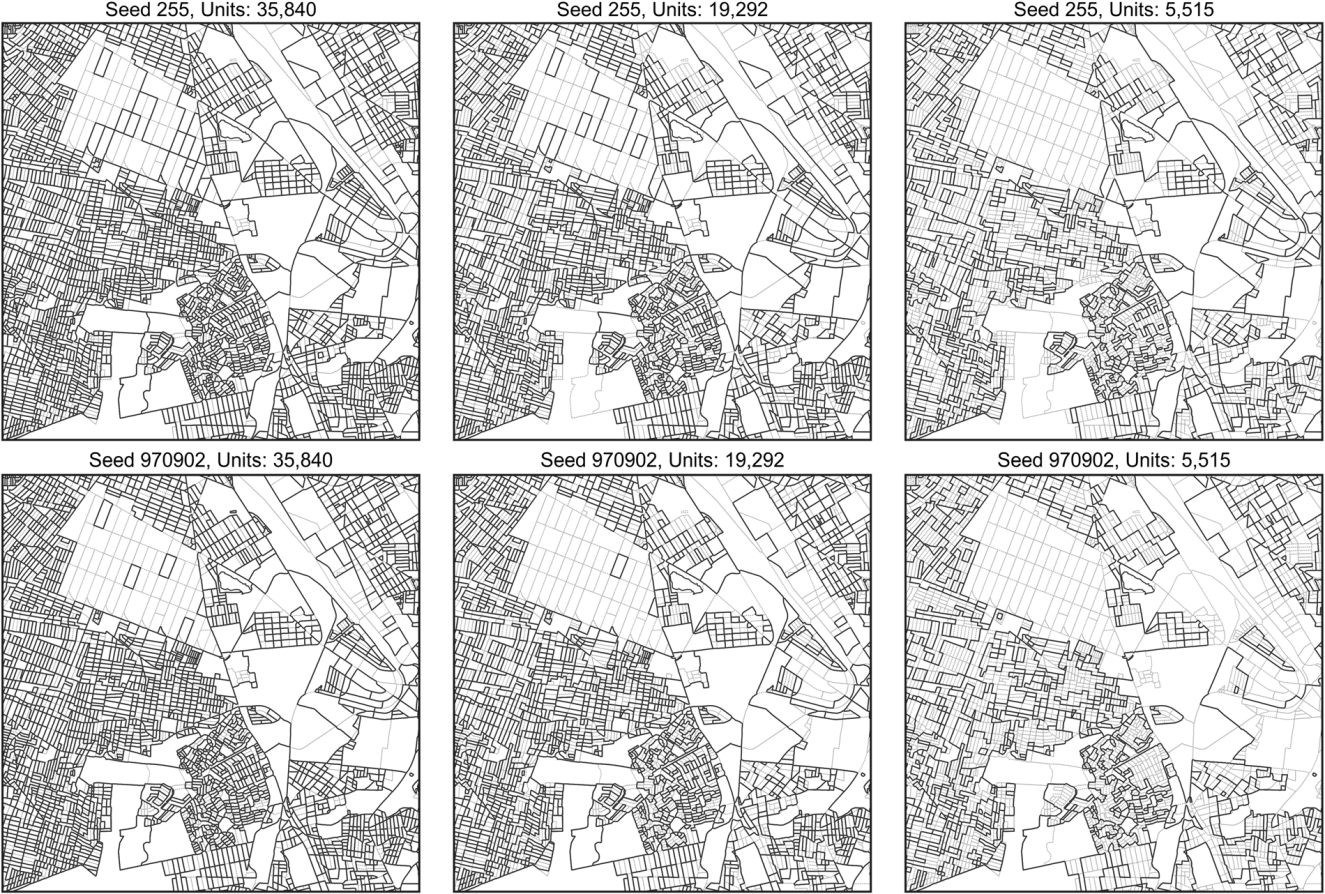
Example of the changing boundaries of the spatial units, in central Guadalajara, as a given simulation seed progresses from the original 55,146 unit boundaries (lighter grey lines) to target, merged unit boundaries (darker lines). Two random seeds are shown here for selected numbers of target units to show relative progression from 55,146 to 5,515 units.

For computational efficiency, and given the correlative interdependency between runs of a given seed, from here we only examine the data corresponding to the 95, 85, 75,…, 15, 10 percent datasets. Prior to the described aggregation procedure, the fine-scale data, i.e., validation population, needed to be pre-processed for this task. An example of the progressive coarsening process is shown in Fig. 4 and demonstrates the variation in the merging between seeds.

All simulation computation was done utilising R Statistical Software v. 4.1.0^54^ and the packages^55–63^ indicated in the provided code notebook. It took 567 hours of computation to produce the simulated datasets, with each job utilising one core and 9.5GB RAM of a standard core on the Barkla High Performance Computing (HPC) environment at the University of Liverpool (https://www.liverpool.ac.uk/it/advanced-research-computing/facilities/high-performance-computing/). The jobs were run in parallel across seeds, but sequentially for each five-percent decrease in the number of units for each seed (Fig. 3).

## Data Records

The simulated data^47^ produced using the aforementioned procedures is stored at in a Harvard Dataverse Data Repository (10.7910/DVN/XBKPLE) and contains four folders: Merge_Logs, Original_Units, Simulated_Units, and Supplementary.

### Merge_Logs

This compressed folder contains a single.RDS file holding a R data.frame object. Here, each row corresponds to the merge of areal units in a given iteration of the simulation (Fig. 3) and is composed of columns that record the simulation seed, iteration counter, the target number of units of the simulation, the unique ID of the areal unit that was merged and the unique ID of the areal unit it was merged with (and was relabelled as). From this data.frame, it is possible to retrace the sequence of areal unit merging and even represent this as a network diagram.

### Original_Units

This compressed folder contains two folders, both containing a single shapefile containing polygons representing our areal units. The Street_Gapped folder contains the original census-based units with no data where streets lay within more densely populated areas. The Tessellated folder contains the same data after it went through the momepy processing, extending the areal boundaries to fill in the street gaps. The Street_Gapped and Tessellated folder data correspond to the left and right panels of Fig. 1, respectively.

### Simulated_Units

This folder contains nine folders, each corresponding to a set of 100 simulated areal units of a given number of units (Fig. 3), as indicated in the folder name “Units_ < no. of areal units > ”. These folders contain a number of compressed archives that can be unzipped utilising free software such as 7-zip (https://7-zip.org/) or tools such as the R archive package^64^. Within these archives are the simulated areas in Shapefile format. The archives within the folders are all below 2.5GB in size (when compressed) to comply with repository limits and to are provided for individual download as many users will not want to utilise the entire collection.

Within each archive are shapefiles with each of these shapefiles corresponding to a unique random seed utilised to facilitate the merging process to produce the simulated data sets. There are 100 shapefiles for each folder, totalling 900 shapefiles overall. The shapefiles adopt the following structured naming convention indicating the parameters of the creation of the simulated data.

“MEX_admin_SIMULATED_Aggregation_seed_ < random seed value > _scale_ < scale value used in probabilities > _target_ < no. of areal units > .shp”

Each shapefile contains four columns, corresponding to each feature’s: 2010 population count (P2010), area in km^2^ (AREA), the corresponding population density (POP_DENS), and the unique geographic ID (GUBID_INT).

### Supplementary

This folder contains a single compressed folder titled Unit_Frequency_In_Simulations. Within this folder, are two files: a shapefile, with the original 55,146-unit boundaries, containing information on how often the individual features are present across all target values in the 1000 simulations of coarsened data and a README.txt file describing the shapefile data.

The shapefile should be utilised by end users to understand how many simulations a given, individual areal unit was merged with *at least* one another unit. Of particular use would be the creation of choropleth maps where the number or percentage of simulations for a given target value are mapped to the colour scale.

This is important for inclusion/exclusion of error metrics calculated in units when assessing end use impacts or unit scale and zonation. For instance, if looking at calculating error metrics for modelled population in the area covered by the original unit ID “XXXXXX” for target value 5515, and the choropleth map shows that, across all 100 simulations, this unit was merged with one or more unit in only four of those simulations. A user would want to exercise more caution in the robustness of error metrics, particularly in comparison another unit which may have been merged with one or more units in, say 90 of 100 simulations. This is particularly so when trying to create non-parametric bootstrapped estimations of confidence intervals or similar procedures as, following the above example, one of these would be created with an effective sample of four versus another unit being created with an effective sample of 90.

## Technical Validation

The following serves not only as a technical validation of the dataset^47^, but also a practical one with the simulated data used to produce dasymetrically modelled gridded population data. Given the described simulation procedure, for simulated population counts we would expect a rightward shift in the distribution of values, i.e., increase in unit population count values, as we decrease the number of units, given that we are summing the counts during our merging processes. We would also anticipate that the number of units with population counts of zero would approach zero as the number of units decreases due to the same summation process.

For simulated areas, we would expect a decrease in the near zero values due to the quasi-random sampling process that increased the probability of selecting smaller units for merging, along with a general rightward shift in the distribution of values. Related to these, we would expect that the changes in population density distributions to be some combination of these shifting distributions, by definition. However, due to our merging process, which selected the neighbouring unit with the least difference in population density, the shifts in the general shape of the population density distributions are minimised.

To interrogate if the simulated areal units presented here behaved as expected, and to ensure that they are fit for further modelling and analytical purposes, we produce a few brief case studies of RF-informed dasymetrically disaggregated gridded population surfaces. This procedure disaggregates areal population counts to smaller spatial units within each source area, utilising weights generated by a RF regression trained at the source unit level and using environmental covariates^38^. To do so, we utilise the popRF package^65^

**Fig. 5:**
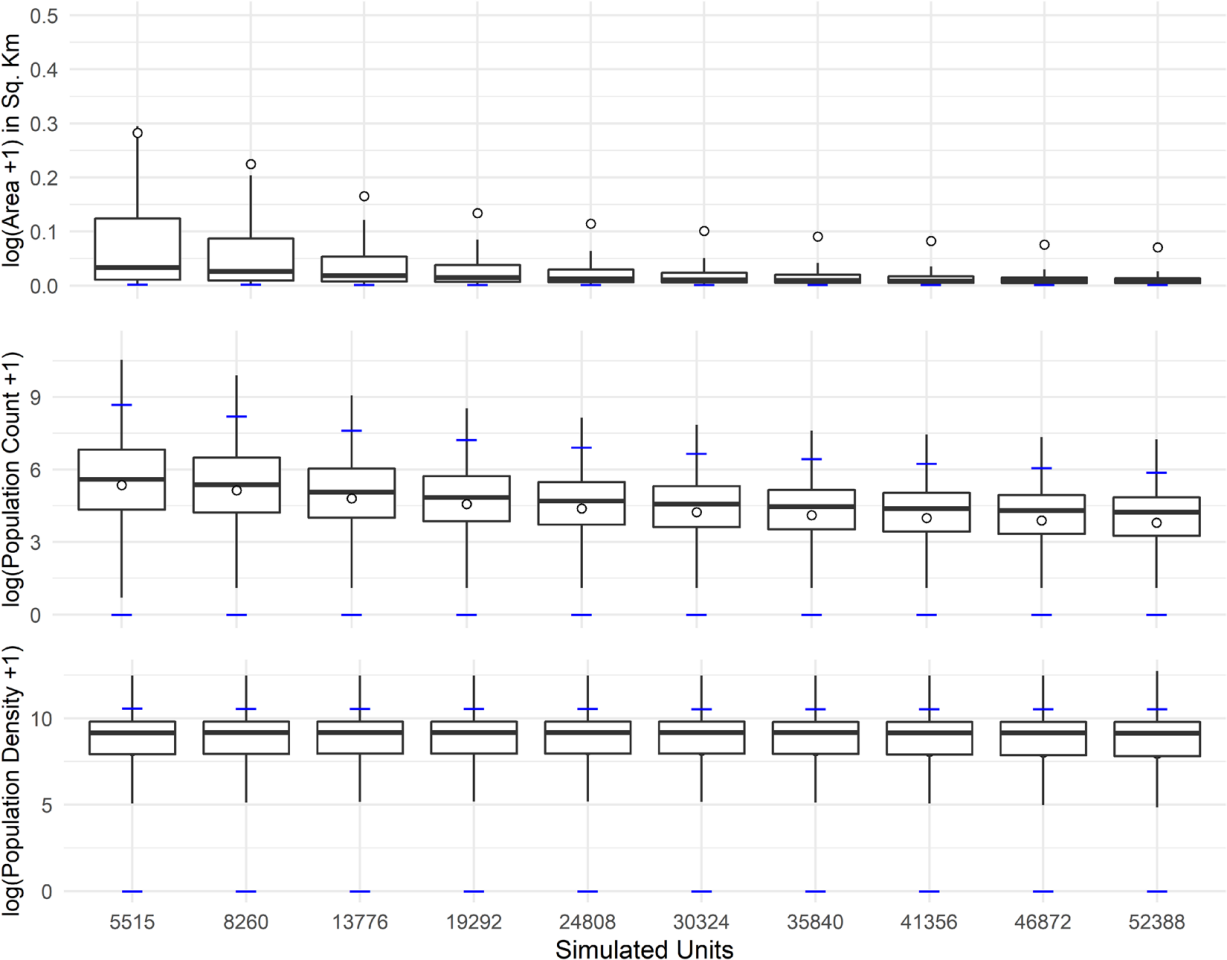
Box plots of the log transformed values of the simulated unit areas and corresponding population counts and population densities, at specific target units. Each boxplot is composed of 100 simulations each using a unique random seed. The median is given by the bold black line and the mean given by a white circle (off plot boundaries for population density). The 2.5th and 97.5th percentiles are given by the blue horizontal lines (off plot boundaries for area).

We can see in Fig. 5 that our assumptions for the simulated data were met. In all, for area and population count, we see a trend of decreasing median and mean values as the number of units decreases. For area, we also see a corresponding decrease in variability with decreasing number of units, and a similar, more muted decrease in variability of population counts. The largest finding here is just how effective our merging process was at retaining the overall range of population density values (bottom panel, Fig. 5). There is very little change in the shape and spread of the distribution of population density values. In the context of dasymetric disaggregations of population counts as informed by statistical means, this is important because it preserves much of the variance, i.e., information, for the weights producing model to train upon while still increasing the variance of population counts and areas where the weights will be used to redistribute the data.

**Fig. 6:**
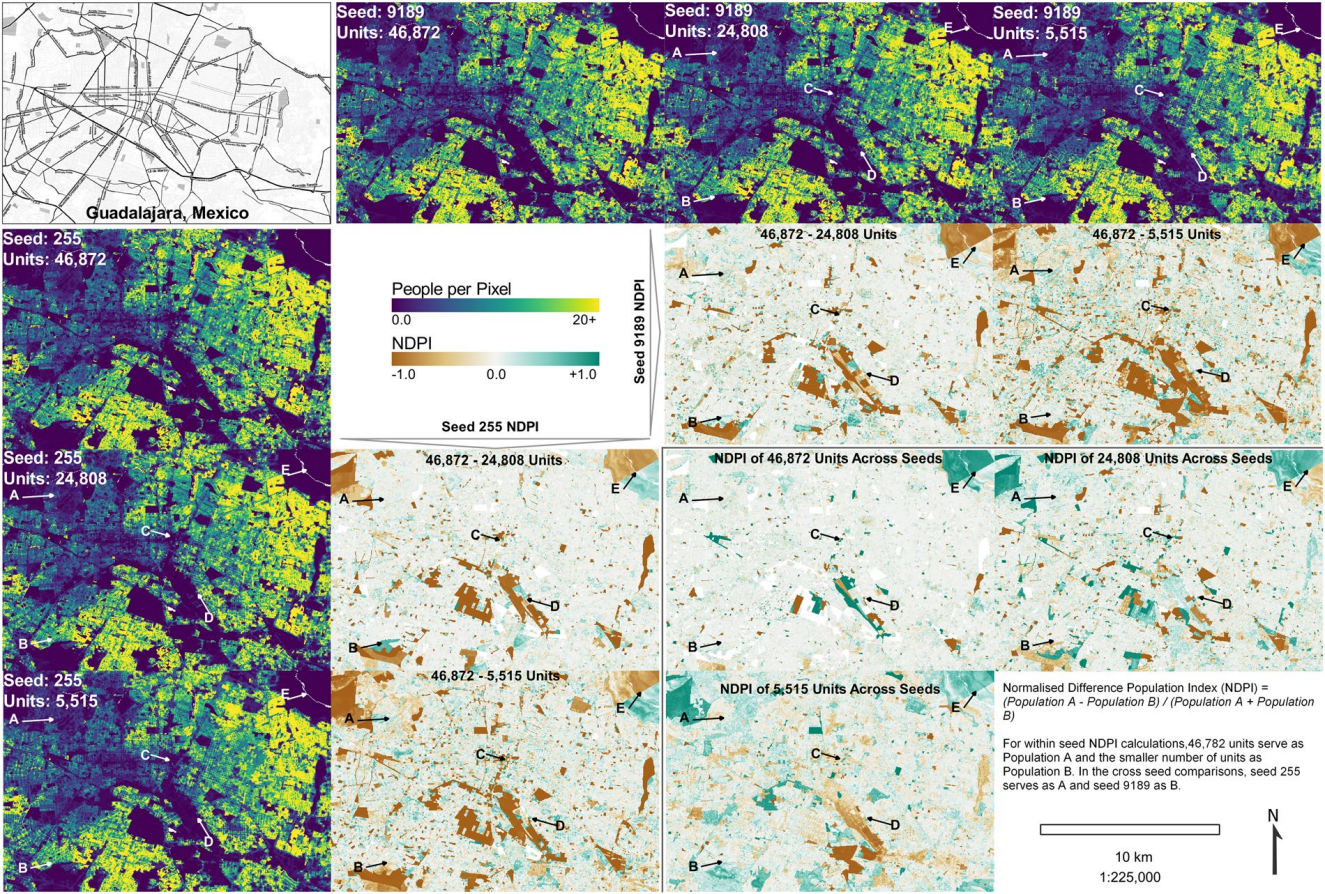
Brief comparison of RF-informed dasymetrically distributed populations using different realisations of the simulated data – between two seeds and three different amounts of simulated units. The Normalised Difference Population Index (NDPI) of these population rasters is shown within and between seeds.

For our limited population modelling example, we selected two random seeds and looked at three target values from across the range of target values available (Fig. 6). Examining the people per pixel (ppp) subfigures, we can only see subtle visual differences in the distributions of values for a given number of target units. As we look at the same ppp subfigures across the range of target units for a given seed, we start to see more obvious differentiation which could be generally described as an increased spatial smoothing with the decreased number of units. These differences, both across target units and across seeds, become more apparent when looking at the Normalised Difference Population Index (NDPI) which, like the more common Normalised Difference Vegetative Index (NDVI), treats differences in values at both low and high magnitudes with equal weight. NDPI is calculated as shown in Eq. 2.

2 $$NDPI=\frac{\left(Population\,A-Populatio\,B\right)}{\left(Population\,A+Populatio\,B\right)}$$

When examining between the highest number of target units and the relatively lower number of units within a single seed, we see the pattern of the largest negative and positive differences, where the model, informed by less units, overestimates the population relative to the model informed by a greater number of units, occurring in areas of lower population (Fig. 6, Points A - E). These also happen to occur in the larger spatial units, which are known to be correlated to low population areas (Fig. 6, Point E). These large magnitude NDPI areas increase in both magnitude and frequency as the number of target units decreases. This is to be expected, as the size of the source unit, to use the dasymetric nomenclature from^30,31^, increases, so does the spatial uncertainty in any disaggregation simply due to the greater number of potential target units to distribute the count values to.

Looking at the NDPI values when holding the number of target units constant and between two seeds (Fig. 6, bottom right), we can see that there are differences, again occurring with the highest magnitude in the largest and least populated units (Fig. 6, Points C - E). These differences increase between seeds as the number of target units decreases, in part due to spatial uncertainty in disaggregation but also due to greater simulation path divergence as more units are merged using different random seeds.

## Usage Notes

The data^47^ and their production methodology are presented here with dual purposes in mind. The first is to provide a common set of synthetic data, produced across an entire domain of realistic levels of spatial aggregation, usable in a diverse array of spatial and process-based modelling approaches. The second is to provide a common methodology packaged in the form of code and example usage that can be used to produce such areal unit data in contexts outside the Mexican subset and the synthetic datasets we provide. The most important aspect of both data and methodology here is the choice to conserve a feature of interest, such as population density, across levels of spatial aggregation.

With regards to the use of the finest level data for comparison against disaggregated or modelled data from coarser, synthetic versions, a key piece of metadata to rely on is that of how many times each original unit has been coarsened (refer to shapefile in Supplementary Data). This information can be leveraged to subsample data from the finest level for various uses (e.g. choose those original units that have been used frequently, or vice versa). This approach was illustrated in our use case scenario, which shows how repeat modelling simulations across various realisations of the aggregated data can produce bootstrapped, fine (e.g. pixel-level) prediction intervals in the context of disaggregation or other types of small area estimation.

We argue that these simulations, and the methods to produce them, are most useful for assessing the zonation and aggregation effects that are present in real-world data where areal units can be variable in size, shape, or character. The effects of incorporating such areal data into modelling and analyses at various levels of aggregation can sometimes be opaque and incorporating systematically aggregated levels of data for analysis can produce better predictability of these modifiable areal unit effects. With regards to population disaggregation modelling applications, this dataset is best suited for understanding the spatial sensitivity of a model to the number of units used for training and the effect of spatial resolution of areal units in the spatial uncertainty induced through disaggregation. It is not well suited for understanding how changing areal units, through coarsening, vary with population densities due to our density preserving merging selection criteria (Fig. 3, bottom panel). Such a simulated dataset would be desirable for understanding how changing distributions and ranges of input population densities then affect model training outcomes and predictions, but would require a modification of the procedure to merge a selected unit randomly or by maximising the population density difference with the selected neighbouring unit to be merged with.

## Data Availability

The code utilised in producing this dataset was originally a series of individual scripts in R and, for submitting jobs, to the HPC, in Bash. We have compiled these scripts, including job submission scripts, into a single ordered R notebook to ease comprehension and replicability^66^. All packages indicated in the notebook utilised the most recent version available on November 1, 2021. The code notebook is available at the following Github repository release: https://github.com/jjniev01/areal_sandbox.
